# Predicting severity of cartilage damage in a post-traumatic porcine model: Synovial fluid and gait in a support vector machine

**DOI:** 10.1371/journal.pone.0268198

**Published:** 2022-06-08

**Authors:** Jonah I. Donnenfield, Naga Padmini Karamchedu, Benedikt L. Proffen, Janine Molino, Martha M. Murray, Braden C. Fleming

**Affiliations:** 1 Division of Sports Medicine, Department of Orthopaedic Surgery, Boston Children’s Hospital, Harvard Medical School, Boston, MA, United States of America; 2 Department of Orthopaedics, Warren Alpert Medical School of Brown University/Rhode Island Hospital, Providence, RI, United States of America; Drexel University, UNITED STATES

## Abstract

The inflammatory response to joint injury has been thought to play a key role in the development of osteoarthritis. In this preclinical study, we hypothesized that synovial fluid presence of inflammatory cytokines, as well as altered loading on the injured leg, would be associated with greater development of macroscopic cartilage damage after an ACL injury. Thirty-six Yucatan minipigs underwent ACL transection and were randomized to: 1) no further treatment, 2) ACL reconstruction, or 3) scaffold-enhanced ACL restoration. Synovial fluid samples and gait data were obtained pre-operatively and at multiple time points post-operatively. Cytokine levels were measured using a multiplex assay. Macroscopic cartilage assessments were performed following euthanasia at 52 weeks. General estimating equation modeling found the presence of IL-1α, IL-1RA, IL-2, IL-4, IL-6, and IL-10 and MMP-2, MMP-3, MMP-12, and MMP-13 in the synovial fluid was associated with better cartilage outcomes. Higher peak pressure for the surgical hind leg and contralateral hind leg aligned with worse cartilage outcomes. A support vector machine built with synovial fluid and gait metrics also demonstrated cytokine presence was predictive of better cartilage outcomes. In conclusion, this preclinical analysis suggests that synovial fluid devoid of cytokines may be a possible indicator that cartilage is more at risk of becoming pathologic after joint injury.

## Introduction

Nearly 200,000 ACL tears occur each year in the United States [1]. Of the individuals who experience such a tear, about half will develop posttraumatic osteoarthritis (PTOA) in one to two decades [2]. This outcome discrepancy elicits two questions: (1) Why do some individuals with seemingly similar medical histories develop PTOA while others do not? (2) More practically, are there biomarkers that can predict PTOA outcomes following injury so earlier intervention can be initiated? While the first question is undoubtedly important, the second question, being more targeted, is the focus of this study.

Biochemical markers are a longstanding osteoarthritis (OA) biomarker category. In particular, serum and urine biochemical markers (in combination with MR imaging) have been combined to produce promising OA phenotype classification systems [3]. These efforts have led to mounting evidence that urinary CTX-II and serum MMP-1, MMP-3, COMP, and hyaluronan have some predictive value for OA in addition to their classification uses [4]. Beyond these media, there has been growing interest in synovial fluid biomarkers. Although these signatures have been moderately successful in classifying OA burden [4, 5], they failed to predict OA outcomes [6]. However, these synovial fluid studies have shortcomings in that they either had insufficient quantities of most assayed targets, or they relied too heavily on imaging as a proxy for progression of joint disease [5, 6]. Notably, one study found that synovial fluid levels of MMP-3, TIMP-2, and VEGF prior to surgical incision for injury-related arthroscopy moderately predicted patient pain scores five years postoperatively [7]. These inconsistent findings suggest that the predictive capacity of synovial fluid biomarkers has yet to be resolved in the context of PTOA and warrants further study.

In addition to biochemical markers, kinematic features, such as gait, have been linked to PTOA outcomes following injury [8]. However, previous studies have defined outcomes by either imaging or pain/function signs as opposed to direct observation of cartilage integrity. Only recently have more modern techniques, such as machine learning, been employed to find the connections between gait and OA—with most endeavors focusing on classifying healthy and diseased joints as opposed to predicting these outcomes before they occur [9]. In particular, Support Vector Machines (SVM), a machine learning tool that has been invaluable in other areas of medicine such as predicting cancer responses to therapeutics, has been gaining traction in OA research [10–13].

Therefore, we set out to (1) characterize the associations between the presence of synovial fluid biomarkers at multiple early timepoints and posttraumatic macroscopic cartilage damage at 52 weeks in the porcine model, (2) characterize the associations between spatiotemporal gait parameters and posttraumatic macroscopic cartilage damage at 52 weeks, and (3) build Support Vector Machines to assess the individual and combined ability of these variables to predict posttraumatic macroscopic cartilage damage at 52 weeks.

We hypothesized that synovial fluid presence of inflammatory cytokines and MMPs, as well as altered loading on the injured leg, would be associated with greater macroscopic cartilage damage scores when evaluated by generalized estimating equations (GEE). We also hypothesized that SVMs built with either synovial fluid data or gait data would predict cartilage damage scores. Additionally, we posited that combining these datasets would enable the construction of an SVM that exceeds the performance of both individual models.

## Methods

### Animal model

The Institutional Animal Care and Use Committee granted study approval before this experiment was carried out. The study was designed following the ARRIVE guidelines [14]. The study utilized samples from 36 Yucatan minipigs (Sinclair BioResources, Columbia, MO) in late adolescence [age (mean±SD): 15.3±1.6 months; weight: 52.1±4.6 kg]. Each animal underwent unilateral ACL transection and was randomized to one of three experimental groups: no subsequent treatment (ACLT), immediate ligament reconstruction with bone-patellar tendon-bone allograft (ACLR), or immediate ligament restoration using an extracellular matrix scaffold combined with autologous blood (BE-R) as previously described by Karamchedu et al. [15]. This previous study explored surgical group differences in gait metrics (e.g., stance time, stance velocity, maximum force as % of body weight) as well as the effect of surgical group on macroscopic and microscopic cartilage damage and synovitis at 52 weeks post-euthanasia [15]. An additional analysis by Karamchedu et al. 2021 explored surgical group differences in synovial fluid cytokine levels [16]. For that analysis, synovial fluid aspirations were performed pre-operatively as well as at 1-, 4-, 12-, 26-, and 52-weeks following surgery [16]. The current study builds on these two endeavors by combining their respective gait, synovial fluid concentrations, and macroscopic cartilage damage score data to create prognostic machine learning models that predict cartilage outcomes. Details regarding animal husbandry, pain management, and the IACUC-approved surgical procedures have been previously reported [15] and can be found in **S1 Appendix**. Investigators were blinded to animal group assignments following surgical operation.

### Synovial fluid aspiration

Synovial fluid samples were collected at six timepoints (pre-operative, 1-, 4-, 12-, 26-, and 52-weeks) [16]. Under anesthesia, each pig was prone-positioned with their surgical limb hanging over the side of the table in a 90-degree angle of flexion. A 23-gauge needle, outfitted with a 3 ml syringe, was inserted through the patellar tendon to collect synovial fluid from the femoral notch region. Aspiration occurred under slight negative pressure. If unsuccessful, the draw was repeated after injection of 10 cc of sterile phosphate buffer saline. Following aspiration, the collected synovial fluid underwent centrifugation at 1300 RCF, and the supernatant was stored in 50 μl aliquots at -80 degrees Celsius. If the aspiration was unsuccessful, the collection was repeated after injecting an additional 10 cc of phosphate buffered saline. During the same procedure, 10 ml of blood was collected from the cranial vena cava and portioned into two 5 ml vacutainer serum separation tubes. Serum separated at room temperature for 30 minutes before being centrifuged for 15 minutes at 3000 RCF. The supernatant was stored in 1.5 ml aliquots. Serum samples were used to calculate synovial fluid dilution as described in the next section [17].

### Multiplex assay

Samples were thawed at room temperature for an hour and a half. A custom multiplex assay kit (SPR#1178, Millipore, Burlington, MA) was used to assess the concentrations of 19 target proteins, 8 matrix metalloproteinases (MMPs) and 11 cytokines (**Table 1**) as previously described [16]. Each synovial fluid sample was assayed twice, and the average value was used. Multiplex technology (Bioplex-200; BioRAD, Hercules, CA) was used to measure fluorescent intensity. Fluorescent intensities and concentrations of standards were used to establish a standard curve. It should be noted that cytokine fluorescent intensities were considered “detectable” if they had concentrations above their lowest standard. Therefore, each cytokine had a different lower limit of detection, which ranged from 2.44 pg/mL (for MMP-9) to 94.7 pg/mL (for MMP-7). Mean fluorescent intensities served as input for 5PL logistic regressions to obtain concentration estimates. Concentration estimates were then averaged between duplicates using commercial software (Bioplex Manager; BioRAD, Hercules, CA). For each sample, the ratio of synovial fluid urea concentration (post-averaging) to serum urea concentration was obtained using a blood urea nitrogen (BUN) assay (ab83362, Cambridge, MA) [17]. Concentrations are expressed in picograms per milliliter (pg/ml).

**Table 1:** The eight matrix metalloproteinases and eleven cytokines included in this experiment.

MMPs	Cytokines
MMP-1	IL-1α
MMP-2	IL-1RA
MMP-3	IL-2
MMP-7	IL-4
MMP-9	IL-6
MMP-10	IL-8
MMP-12	IL-10
MMP-13	IL-12
	IL-18
	GM-CSF
	TNFα

### Gait metrics

As previously described by Karamchedu et al., gait data were collected using a pressure mat (HRV6 Walkway System; Tekscan Inc, Boston, MA), which had a sensing area of 292.6 x 44.7 cm [15]. Animals were conditioned to walk unidirectionally on the pressure mat with food serving as a reward. Step calibrations were performed for each sensing tile using a custom 58 kg three-legged phantom, as recommended by the manufacturer. Data acquisition was initiated by first hoof contact and continued at 104 Hz until the animal stepped off the mat. Data collection and analyses took place using commercial software (Walkway 7.0; Tekscan Inc, Boston, MA). Gait data were collected at five timepoints (pre-operative, 4, 12, 26, and 52 weeks) with no collection at 1 week because animals were still recovering from surgery [15]. For each timepoint, five gait trials were conducted, and all metrics were averaged. Hoof strikes were detected automatically by the software, and partial strikes were discarded. By recording force and time, we were able to document maximum force (kg) as a percentage of body weight, maximum peak pressure (kPa), and impulse (kg-sec) as a percentage of body weight for each limb. Spatiotemporal recordings allowed us to determine stride length, stride time, stride velocity, and stance time.

### Macroscopic cartilage assessment

After euthanasia and joint harvest at 52-weeks post-surgery, articular cartilage surfaces were assessed for macroscopic damage according to Osteoarthritis Research Society International (OARSI) guidelines for sheep and goat [18]. Damage to six articular surfaces, the medial femoral condyle, medial tibial plateau, lateral femoral condyle, lateral tibial plateau, femoral trochlea, and patella, were scored from 0 (normal) to 4 (large erosions down to subchondral bone). These scores were then summed to create a macroscopic score that ranged from 0 to 24.

### Statistical analyses

All statistical analyses were carried out in R version 4.0.1 [19]. Pre-operative synovial fluid and gait data were excluded from the analysis so that predictions would solely be based on post ACL transection data. Synovial fluid data from week 1 were also excluded because no gait data were collected at that time point.

A gaussian mixture model was used to soft cluster the 24-point damage scores into two subpopulations. Subjects were ultimately labeled as having either “good” or “bad” cartilage outcomes based on which subpopulation had greater than a 50% chance of belonging to. Multiplex assay results were binarized by assigning a one if synovial fluid levels were above the lower limit of quantification (i.e., presence) or a zero if the levels were below the lower limit of quantification (i.e., absence). Subjects and synovial fluid targets were then hierarchically clustered based on multiplex results according to the Ward.D2 method, which squares dissimilarities before clustering based on variance minimization [20]. Results were visualized using a heatmap.

Each gait parameter was normalized to have zero-mean and unit-variance. Generalized estimating equations (GEEs) were used (1) to model cartilage damage outcome as a function of target protein presence in the synovial fluid while accounting for within-subject covariance, and (2) to model cartilage damage outcome as a function of each gait parameter while accounting for within-subject covariance. GEEs were used because each subject had synovial fluid sampled at multiple timepoints, which necessitated adjusting for within-subject covariance. A p-value of 0.05 served as the threshold for statements about statistical significance.

### Machine learning methods

Before training the machine learning models, the dataset of 129 synovial fluid and gait profiles was randomly divided 70/30 into a training set (N = 91) and a testing set (N = 38), respectively. The testing set remained untouched until a model had been optimized on the training set.

This study implemented the SVM algorithm, a classical machine learning method that computes a linear hyperplane to geometrically separate labeled points in variable space [21]. The SVM was built in Python (Version 3.7.3) using scikit-learn [22, 23]. The training set was iteratively validated using leave-one-out cross-validation (LOOCV). For feature selection, we first manually built four SVM models: synovial fluid data only, gait data only, synovial fluid and gait data combined, and an SVM that only included features deemed significant by GEE analysis. Second, we implemented our SVM with L1 (i.e., lasso) regularization with its cost function shown here:

$$Cost=\sum_{i=1}^{N}[{y_{i}-\sum_{j=1}^{m}\left(w_{j}\times x_{ij}\right)]}^{2}+(\frac{1}{2NC})\sum_{j=1}^{m}\left|w_{j}\right|$$

*N* is the number of samples, *m* is the number of features, *w* is the weight (i.e., hyperplane coefficient) for each feature, and *C* is the regularization parameter [23]. To further optimize the models, we tested two kernel functions—linear and radial basis function (RBF)—to see which would provide the greatest area under the receiver operating characteristic curve (AUROC). For the linear and RBF kernels, we optimized the regularization parameter, *C*, by iteratively increasingly it by 1.0 in log-10 space and recalculating the AUROC. The RBF kernel required additional optimization of the γ parameter, which was also determined by iterations of 1.0 in log-10 space while holding *C* constant. The optimal models were found using a linear kernel with varying *C* values which are reported in the text. This is analogous to the grid search method used in previous SVM OA prediction models [10].

Performance statistics are reported for both training set and testing set models. Receiver Operating Characteristic (ROC) and Precision-Recall (PR) curves were generated for training and testing sets to gauge sensitivity-specificity tradeoffs and Positive Predictive Value (PPV)-sensitivity tradeoffs (S2-1 and S2-2 Fig in **S2 Appendix**; S3-1 and S3-2 Fig in **S3 Appendix**). Area under the ROC curves and PR curves are reported. SVM models were optimized by greatest AUROC to place equal focus on correctly predicting both good and bad cartilage outcomes.

## Results

### Clustering and data allocation

**Fig 1:**
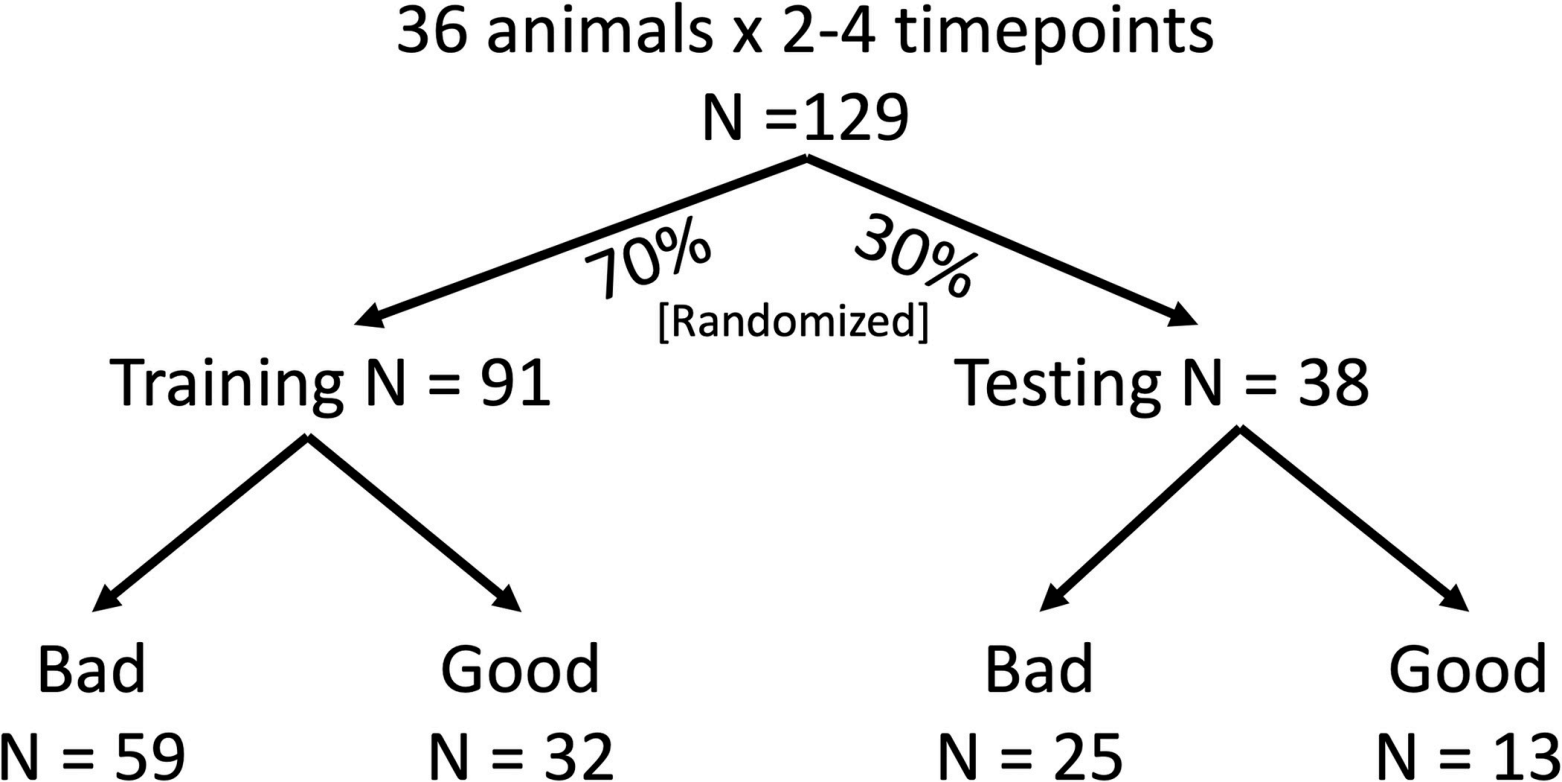
70% of the data were allocated to training and building the SVM models. The remaining 30% of the data remained untouched for later assessment of the trained models. At every level of analysis, there were more “bad” outcome samples than “good” outcome samples.

**Fig 2:**
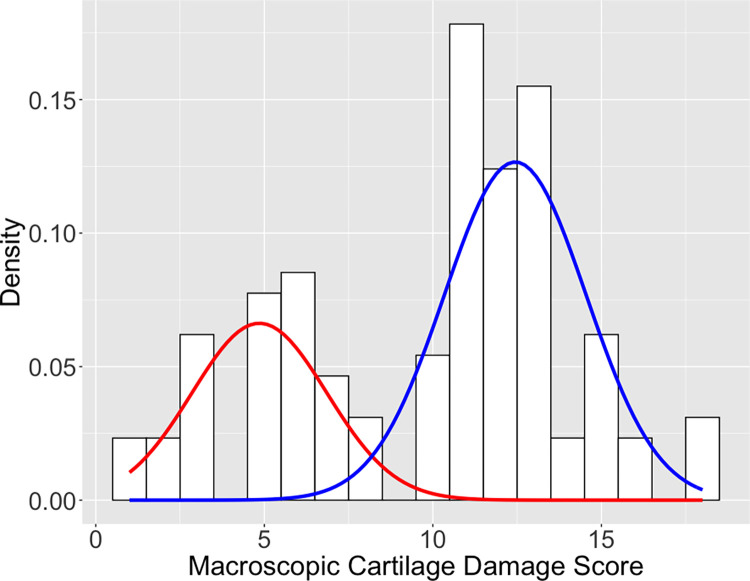
A gaussian mixture model was used to evaluate the presence of two clusters of cartilage damage outcomes. The red and blue lines represent the different gaussian distributions for “good” and “bad” outcomes, respectively. The optimal delineation between the two damage outcomes is between scores 8 and 9.

A gaussian mixture model identified the presence of two subpopulations of damage score outcomes on the 24-point macroscopic cartilage damage scale (**Fig 1**). Scores ranged from 1 to 18. Damage scores were assigned to the cluster for which they had greater than a 50% chance of belonging to. The optimal delineation between subpopulations fell between scores 8 and 9 (i.e., scores 8 and below were classified as “good” outcomes, and scores 9 and above were classified as “bad” outcomes). The full breakdown of cartilage sub-scores for the “good” cartilage and “bad” cartilage groups can be found in the **S4 Appendix**. Of the 129 total synovial fluid profiles, 48 were in the less damaged (“good” cartilage) group, and 81 were in the more damaged (“bad” cartilage) group. To ensure that training and testing sets had equal proportions of good and bad outcomes, the 129 profiles were randomized with 70% of profiles going into the training set, and 30% of profiles going to the testing set (**Fig 2**).

**Fig 3:**
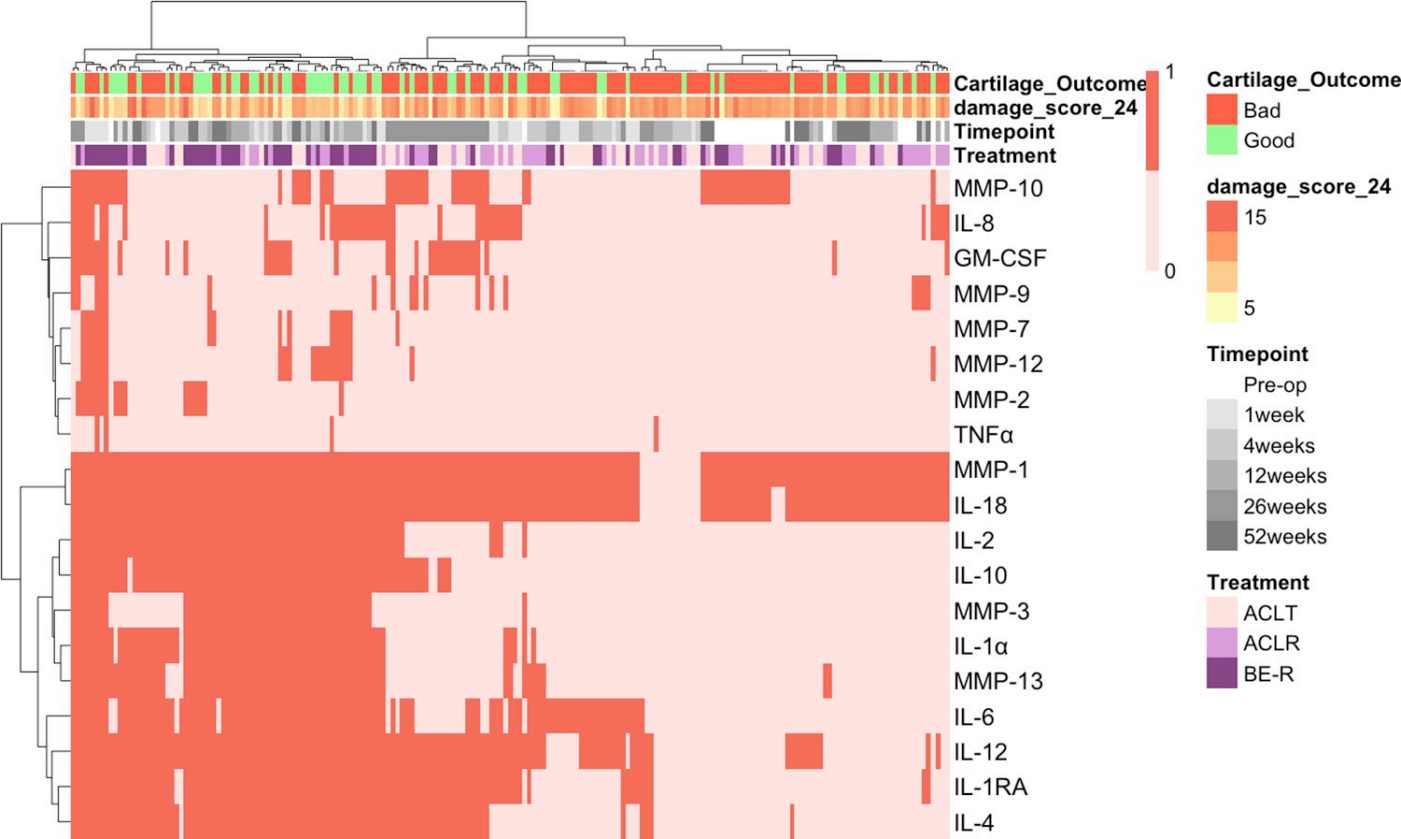
Heatmap of cytokine and MMP presence in the synovial fluid samples. Darker color indicates presence; lighter color indicates absence. Rows and columns are hierarchically clustered according to Ward.D2. Binary and 24-point scale cartilage outcomes are depicted as well as each subject’s treatment and time of synovial fluid aspiration.

Hierarchical clustering of subjects and synovial fluid targets produced a heatmap with qualitative evidence of separation between the outcome groups (**Fig 3**). Moreover, the two largest clusters in the subject dendrogram featured qualitatively distinct synovial fluid profiles—with “good” outcome subjects often having greater presence of the various cytokines and MMPs.

### Generalized estimating equations

For the majority of the protein targets, presence in synovial fluid was associated with “good” cartilage outcomes (**Table 2**). Specifically, cytokines IL-1α (OR = 3.9; P < .001), IL-1RA (OR = 2.5; P = .024), IL-2 (OR = 3.3; P = .002), IL-4 (OR = 3.0; P = .006), IL-6 (OR = 2.6; P = .017), and IL-10 (OR = 3.1; P = .004) were associated with less articular cartilage damage at 52 weeks. Similarly, presence of MMP-2 (OR = 2.2; P = .008), MMP-3 (OR = 3.4; P = .002), MMP-12 (OR = 6.8; P = .006), and MMP-13 (OR = 3.4; P = .002) were each associated with better cartilage outcomes. In the GEE analysis, no cytokines or MMPs were negatively associated with “good” outcomes.

**Table 2:** Linear model parameter estimates.

Biomarker	Coefficient	OR [95% CI]	P-value
**GM-CSF**	-0.01482	0.9853 [0.3962, 2.45]	.975
**TNFα**	-38.95	1.209e-17 [0, Inf]	.999
**IL-1α**	1.35	3.856 [1.782, 8.345]	< .001*
**IL-1RA**	0.9163	2.5 [1.131, 5.528]	.024*
**IL-2**	1.208	3.346 [1.564, 7.16]	.002*
**IL-4**	1.107	3.025 [1.368, 6.689]	.006*
**IL-6**	0.938	2.555 [1.184, 5.513]	.017*
**IL-8**	0.5693	1.767 [0.7126, 4.381]	.219
**IL-10**	1.131	3.099 [1.449, 6.628]	.004*
**IL-12**	0.4109	1.508 [0.6252, 3.639]	.360
**IL-18**	1.892	6.63 [0.814, 54]	.077
**MMP-1**	1.892	6.63 [0.814, 54]	.077
**MMP-2**	2.182	8.865 [1.772, 44.35]	.008*
**MMP-3**	1.238	3.449 [1.553, 7.656]	.002*
**MMP-7**	0.6806	1.975 [0.5346, 7.296]	.307
**MMP-9**	0.1719	1.187 [0.3611, 3.905]	.777
**MMP-10**	0.1978	1.219 [0.5376, 2.763]	.636
**MMP-12**	1.91	6.75 [1.707, 26.7]	.006*
**MMP-13**	1.23	3.421 [1.594, 7.341]	.002*

GEEs account for within-sample covariance and assess connection between synovial fluid target presence and “good” cartilage outcome. Significant findings (P < .05) are denoted with an asterisk. OR = odds ratio.

Of the gait metrics, only those related to maximum peak pressure (kPa) were associated with better outcomes (**Table 3**). In particular, higher peak pressures for the surgical hind leg (OR = 0.44; P < .001) and contralateral hind leg (OR = 0.59; P = .010) were associated with worse cartilage outcomes.

**Table 3:** Linear model parameter estimates.

Gait Parameter	Coefficient	OR [95% CI]	P-value
**Stride length (hind)**	0.1766	1.193 [0.8262, 1.723]	.346
**Stride length (contra)**	0.2365	1.267 [0.8748, 1.835]	.211
**Stride length (ratio)**	-0.1918	0.8255 [0.5634, 1.209]	.325
**Stride velocity (hind)**	0.2098	1.233 [0.8494, 1.791]	.270
**Stride velocity (contra)**	0.2268	1.255 [0.8632, 1.824]	.235
**Stride velocity (ratio)**	-0.1099	0.8959 [0.6202, 1.294]	.558
**Max pressure (hind)**	-0.8241	0.4386 [0.2839, 0.6776]	< .001*
**Max pressure (contra)**	-0.5225	0.593 [0.3995, 0.8803]	.010*
**Max pressure (ratio)**	-0.381	0.6832 [0.4526, 1.031]	.070
**Max force BW% (hind)**	0.1278	1.136 [0.7855, 1.644]	.497
**Max force BW% (contra)**	0.2399	1.271 [0.8801, 1.836]	.201
**Max force BW% (ratio)**	-0.04874	0.9524 [0.6613, 1.372]	.793
**Impulse BW% (hind)**	-0.05411	0.9473 [0.6555, 1.369]	.773
**Impulse BW% (contra)**	-0.05299	0.9484 [0.6549, 1.373]	.779
**Impulse BW% (ratio)**	0.05266	1.054 [0.7286, 1.525]	.780

GEEs account for within-sample covariance and assess connection between gait metrics and “good” cartilage outcomes. Values in red highlight significant findings (P-value < 0.05). OR = odds ratio, Max = maximum, BW% = percent body weight.

### Models and predictive performance

TNFα was excluded from SVM construction because the training set failed to include any synovial samples with TNFα present. All samples with TNFα presence had been randomized to the testing set, and only a few samples had TNFα presence to begin with, so having none in the training set was statistically likely.

**Fig 4:**
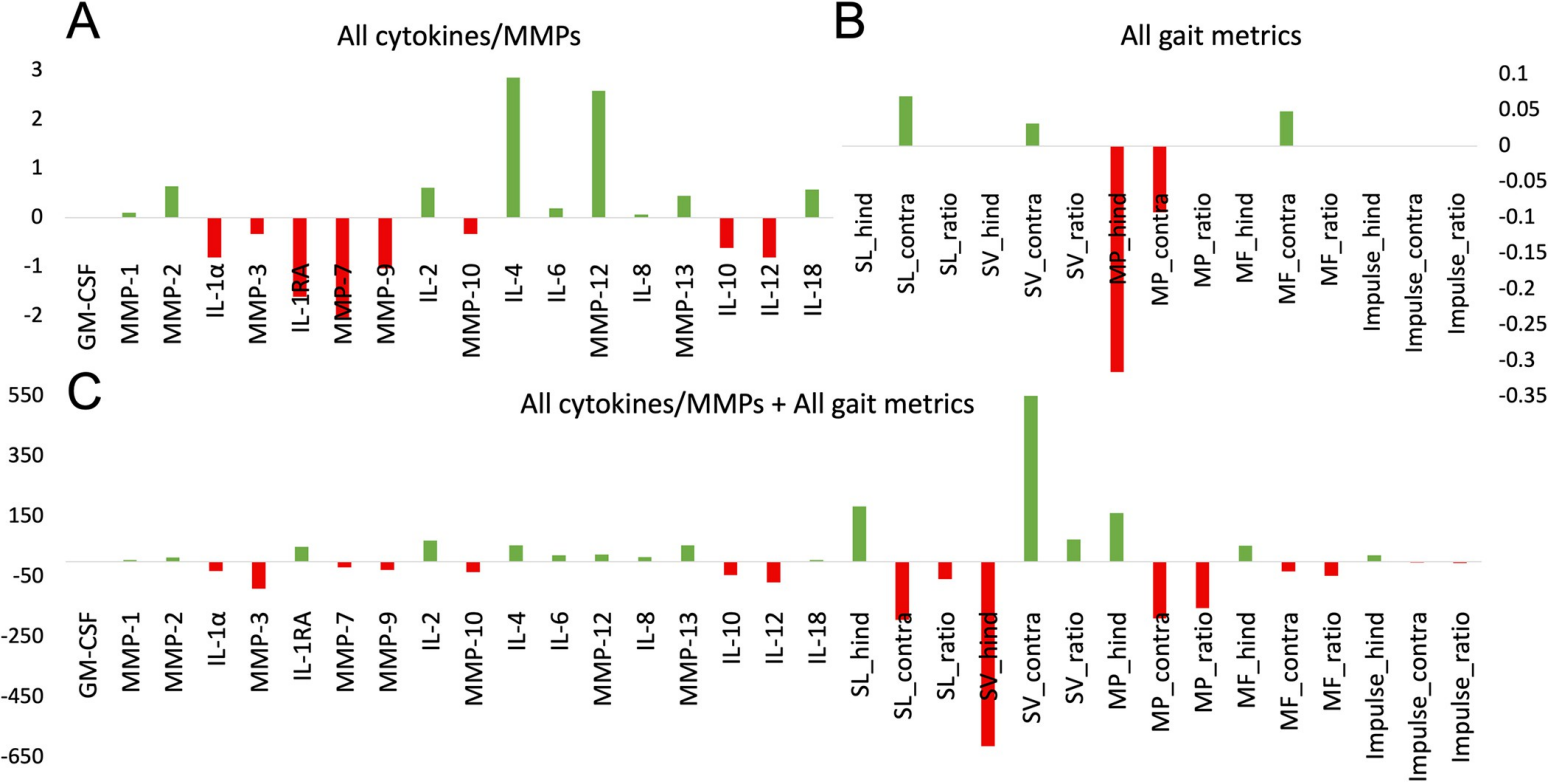
Model coefficients for three Support Vector Machines: (A) synovial fluid-only, (B) gait-only, and (C) the combination of synovial fluid targets and gait metrics.

The SVM built solely with synovial fluid data (AUROC = .609) featured the lowest performance statistics in every category except for specificity when compared to the other three models (**Table 4**). Coefficients for the hyperplane built by the synovial fluid only SVM were roughly evenly divided in terms of being positive (i.e., predictive of “good” cartilage) and negative (i.e., predictive of “bad” cartilage) (**Fig 4A**). Negative predictive value (NPV) and specificity were the top performance statistics for this model, which suggest this model better predicts “bad” cartilage outcomes.

**Table 4:** Training performance statistics for the four support vector machines built in this study.

Training Model	AUROC	AUPRC	Accuracy	Precision	Recall	NPV	Specificity
**Synovial Fluid (C = 1000.0)**	.609	.502	.615	.435	.313	.676	.780
**Gait (C = 0.1)**	.689	.585	.714	.636	.438	.739	.864
**Synovial Fluid + Gait (C = 10000.0)**	.778	.698	.670	.525	.656	.784	.678
**GEE (C = 1.0)**	.752	.694	.736	.700	.438	.746	.898

Each model was optimized to have the highest area under the receiver operating characteristic (AUROC) possible, based on the regularization parameter and L1 feature selection. AUPRC = area under precision recall curve, NPV = negative predictive value.

The SVM built solely with gait data (AUROC = .689) outperformed the synovial fluid-only model (**Table 4**). Following L1 feature selection, the maximum peak pressure of the hind surgical leg and contralateral leg contributed the most to prediction (**Fig 4B**). All positive coefficients were related to the contralateral leg, and the only surgical leg coefficient (maximum peak pressure) was related to “poor” outcomes. NPV and specificity were the top performance statistics for this model which suggests this model better predicts “bad” cartilage outcomes (**Table 4**).

The SVM built with all synovial fluid targets and all gait metrics (AUROC = .778) featured the highest performance in four of the seven categories collected (**Table 4**). Compared to the synovial fluid-only model, the combined model maintained a coefficient direction for the following: MMP-1, MMP-2, MMP-3, MMP-7, MMP-9, MMP-10, MMP-12, MMP-13, IL-1α, IL-2, IL-4, IL-6, IL-8, IL-10, IL-12, IL-18, and GM-CSF (**Fig 4C**). Between the SVM models, only IL-1RA switched direction between the models. Compared to the gait-only model, the combined model changed the direction of most coefficients. In the combined model, the coefficient most predictive of “good” outcomes was higher stride velocity of the contralateral hind leg while the coefficient most predictive of “bad” outcomes was higher stride velocity of the hind surgical leg. NPV and AUROC were the top performance statistics for this model.

**Fig 5:**
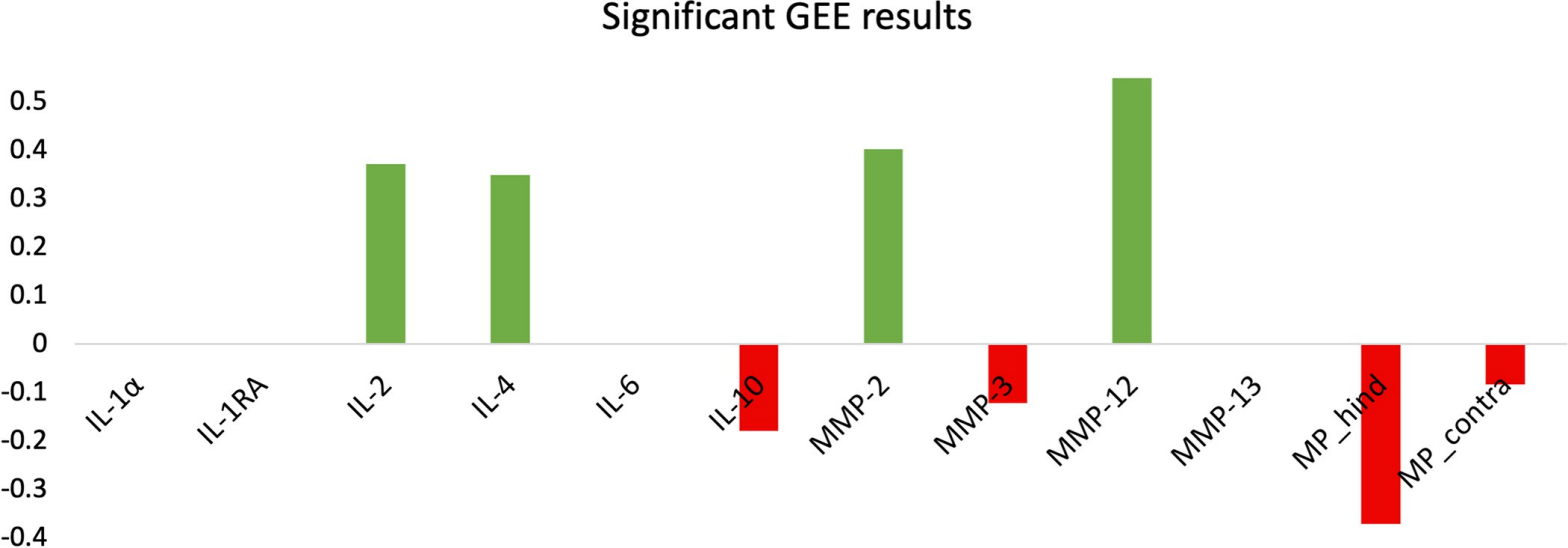
Model coefficients for support vector machine composed of significant features from Generalized Estimation Equation (GEE) analysis.

The SVM built solely with significant features from GEE analysis (AUROC = .752) featured the highest performance in three of the seven categories collected (**Table 4**). Presence of IL-2, IL-4, MMP-2, and MMP-12 were predictive of “good” cartilage outcomes while presence of IL-10 and MMP-3 were predictive of “bad” outcomes (**Fig 5**). Higher maximum peak pressures of the hind contralateral leg and hind surgical leg were predictive of “bad” outcomes with the latter being the most predictive. AUROC and specificity were the top performance statistics for this model. The ROC curves, PR curves, and confusion matrices are reported for each model’s training (S2-1-S2-3 Fig in **S2 Appendix**).

Upon testing, every model featured a decrease in AUROC, though all models but the synovial fluid-only model had an AUROC that remained higher than 0.6 (**Table 5**). Similar to the training performances, NPV and specificity were consistently among the top performance statistics for each model. The ROC curves, PR curves, and confusion matrices are reported for each model’s testing (S3-1-S3-3 Fig in **S3 Appendix**).

**Table 5:** Testing performance statistics for the four support vector machines built in this study.

Testing Model	AUROC	AUPRC	Accuracy	Precision	Recall	NPV	Specificity
Synovial Fluid (C = 1000.0)	.518	.443	.605	.333	.154	.656	.84
Gait (C = 0.1)	.637	.533	.632	.462	.462	.72	.72
Synovial Fluid + Gait (C = 10000.0)	.618	.460	.632	.462	.462	.72	.72
GEE (C = 1.0)	.665	.480	.605	.429	.462	.708	.68

AUROC = area under the receiver operating characteristic, AUPRC = area under precision recall curve, NPV = negative predictive value.

## Discussion

Although we hypothesized that increased presence of inflammatory cytokines and MMPs would be associated with worse outcomes, we found that increased presence of anti-inflammatory markers (e.g., IL-2 and IL-4) and matrix metalloproteinases (e.g., MMP-2 and MMP-12) were associated with better outcomes in both GEE analysis and SVM prediction. With these synovial fluid cytokines and MMPs, we constructed SVMs with satisfactory NPVs and specificities while maintaining adequate AUROCs. Incorporating gait metrics resulted in improved model performance and combining synovial fluid and gait features often resulted in the best predictive performances, which affirmed our last hypothesis. Applying these models to an untouched testing set maintained good prediction of worse quality cartilage. Other cytokines, MMPs, and gait metrics were significant in some models, but not others, and this lack of total concordance, particularly across the gait utilizing SVMs, means that further studies are warranted to verify their predictive utility.

Synovial fluid findings in this study add to the growing narrative that inflammatory markers in the synovial fluid are tied to advancing cartilage disease. For instance, every SVM model that utilized synovial fluid data applied a positive weight to the anti-inflammatory cytokines IL-2 and IL-4—indicating their increased presence as predictive of good cartilage outcomes—and a negative weight to the anti-inflammatory cytokine IL-10. The IL-2 finding goes against a prior study which found an inverse relationship between IL-2 and arthroscopy-visualized cartilage integrity [24]. However, this prior study only had 4 subjects in its less damaged group, and cross-sectionally assessed synovial fluid at a single time point (the end point), while our longitudinal study had higher numbers and used multiple time points prior to cartilage assessment to make its prediction. The IL-4 finding is in accordance with prior studies that found IL-4 to be associated with less osteoarthritic cartilage, and the IL-10 finding is consistent with prior reports of IL-10 associating with greater OA burden—though, as mentioned before, these prior studies evaluated synovial fluid only at the time of OA assessment and not leading up to the assessment [25, 26]. Lastly, IL-18 levels have also been linked to development of OA [27]. Although the probabilistic GEE evidence for a connection between IL-18 and cartilage outcomes was not as strong as it was for other biomarkers in this study, the tremendous GEE effect size (OR = 6.63; P = .077) suggests that IL-18 might still have a role.

Both the GEE analysis and all SVM models suggested that MMP-2 and MMP-12 presence are predictive of better cartilage outcomes. Prior efforts to evaluate synovial fluid MMP-2 levels in OA samples were inconclusive in that it remains unclear if MMP-2 is a hallmark of better or worse OA outcomes [28]. Regarding MMP-12 there have been attempts to distinguish early OA from late-stage OA using synovial fluid MMP levels, but these attempts did not find MMP-12 levels to be of relevance [29]. Our study, therefore, puts forth a novel suggestion that MMP-12 presence in synovial fluid is positively associated with better cartilage outcomes in subjects with OA.

The GEE analysis also found that synovial fluid MMP-3 was predictive of good outcomes, however, every SVM model applied a negative weight to MMP-3, indicating its increased presence as predictive of poor cartilage outcomes. This is in accordance with a prior study linking synovial fluid MMP-3 to OA symptoms and radiographically evident disease [5]. A more recent study also found a link between synovial fluid MMP-3 at time of surgery and OA symptoms five years out, but this study failed to specify the directionality of the relationship [7]. Additionally, a prior study sampled synovial fluid following ACL injury and measured MMP-3 levels but failed to find an association with OA outcomes 16 years out [6]. Thus, further work is needed to define the role of MMP-3 in predicting cartilage outcomes.

In terms of gait metrics, the prior study with the porcine model showed that maximum pressure, impulse, and maximum force of hind limbs correlate with cartilage outcomes [15]. An additional study, employing neural networks, leveraged features such as ground reaction force to classify OA patients by pain and radiographic findings [30]. In the current study, we combined these two approaches to better understand the potential for machine learning to predict cartilage outcomes based on gait metrics. GEE analysis indicated a negative relationship between maximum pressure placed on the hind legs and cartilage damage outcomes. Notably, maximum pressure placed on the surgical leg had a greater effect size than maximum pressure placed on the contralateral leg. Furthermore, the ratio between surgical leg pressures and contralateral leg pressures also indicated a negative relationship with cartilage outcomes—suggesting that greater offloading of the surgical leg onto the contralateral leg was associated with better cartilage outcomes, though elevated pressure on either hind leg was still unfavorable. Of the three SVM models, which made use of gait metrics, all three applied a negative coefficient to maximum pressure of the hind contralateral leg (i.e., higher pressure is more predictive of bad cartilage), and all but the combined synovial fluid+gait model applied a negative coefficient to maximum pressure of the hind surgical leg. The positive coefficient for maximum pressure of the hind surgical leg in the synovial fluid+gait model is most likely an anomalous finding, considering consistency across the other models. But lack of insight into singular discordant coefficients, such as this, remains a drawback of machine learning algorithms.

Despite this differing coefficient, the synovial fluid+gait SVM model had the best performance in the greatest number of performance statistics, including AUROC, AUPRC, recall, and NPV. The second most successful SVM training model, the model which used only variables deemed significant in GEE analysis, had the top performance in all other categories where the synovial fluid+gait model was not the best. It should be noted that the two best performance statistics in every model always included NPV or specificity (or both)—suggesting that every model is best at predicting poor (rather than good) cartilage outcomes. This finding is bolstered by robust prediction of true negatives as seen in the training confusion matrices (**S3 Appendix**). This robust prediction of bad cartilage outcomes, relative to model prediction of good cartilage outcomes, is likely due to the greater prevalence of damaged cartilage in the dataset. As for the performance on the training set, specificity was the highest performance statistic across all models when applied to the testing set. Combined with high NPVs, this means that not only are predictions of poor outcomes often correct, but also that they are unlikely to go undetected. In terms of implications, the fact that these models excel at predicting bad outcomes is suggestive of their practicality, seeing as that identifying subjects most at risk of deteriorating cartilage would be more clinically useful than predicting which subjects’ cartilage will not deteriorate. Shortcomings in predicting good outcomes suggest that these SVM models would best serve as supplementary rule in tests following a more sensitive rule out test. This would play to the models’ strengths in that their specificities give them a better likelihood of correctly predicting bad cartilage outcomes once good outcomes have been included through first pass with a separate, highly sensitive test.

It should be noted that these models were built to detect a cutoff between cartilage damage scores of 8 and 9. A gaussian mixture model was employed to optimize this cutoff by soft clustering cartilage scores into two different gaussian distributions. Manually adjusting the cutoff used in this analysis resulted in a slight decrease in the AUROC but maintained much of the predictive value that these models offer. A decrease in classification performance is expected when de-optimizing the discrepancies between the groups being classified/predicted.

This study has multiple limitations. Firstly, pigs are quadrupeds while humans are bipeds, so precise patterns in gait and distribution of weight differ between the two species, and exact values noted in this study are unlikely to be generalized to humans. Nevertheless, across species certain features are thought to be conserved, such as relative gait adjustments to loading in the setting of pain or decreased joint stability. As a benefit, the porcine model allows for longitudinal assessment of synovial fluid and formal assessment of osteoarthritic cartilage at a time point that is identical for all subjects. The animal model also allows for controlling study variables that are not possible in human studies. But for sake of medical translation, future work will need to scale the findings from the porcine model to that of the human. In addition, the preclinical model utilized adolescent minipigs which were selected for this study because ACL injuries are most common in adolescent patients [1], and this injury places the patient at risk for posttraumatic osteoarthritis [2]. Translating these findings to adults and to the pathogenesis of idiopathic osteoarthritis should be done with caution. Secondly, the ACL injuries were induced with surgical precision. This contrasts with the reality of human injuries which feature a tremendous amount of variation. Creating different degrees of injury would add diversity to the cartilage outcomes and be a good next step for observing the effects of ACL injury on gait and the joint environment post-surgery. Thirdly, the analyses in this study made use of data from 4-, 12-, 26-, and 52-weeks, with the last timepoint also serving as the time of cartilage assessment. This means that predictions were mostly based on timepoints leading up to harvest and that concurrent synovial fluid and gait data were also included in the machine learning algorithms and GEE analysis. This was done to give the predictive analysis as much data as possible to work with; the aim was to see how well prediction could get with a dataset that was dominated by earlier timepoints. In addition, GEE analysis and SVM models had occasional mismatches in their evaluation of different synovial fluid and gait features. This isn’t so much a study limitation as it is a caveat that no single statistical approach comprehensively captures the contributions of features to cartilage health, and it reinforces the value of applying a diverse mathematical approach to characterizing complex biological systems. A notable difference between regression methods (like GEE) and SVMs is that SVMs do not control for variables when evaluating their contribution to the model. Thus, assessing the contribution of any given variable in an SVM has a considerable qualitative component. Additionally, this study featured a moderate imbalance in the number of subjects with good and bad cartilage—which serves as a possible explanation for why the models are better able to predict poor cartilage outcomes. Lastly, this study did not have a true control group for ACL injury. It is important to note that, prior to surgical intervention, subjects had synovial fluid samples drawn and had their gait metrics assessed. These pre-insult data are incorporated into the current study and can be seen in **Fig 3** as “Pre-op.” Regardless, including a group that did not undergo surgery (and presumably developed less cartilage damage) would broaden the scope of this study by contributing to the diversity of cartilage outcomes.

It is critical to note that the biomarkers in this study came largely from timepoints prior to final cartilage collection, a feature that distinguishes this analysis from previous endeavors and literature. While previous studies have individually associated either gait metrics or synovial fluid markers with OA progression, this study collected both types of data from subjects and leveraged them not only for regression-based GEE analysis but also for functional predictive models of OA outcomes. Unique to this study, radiographic scores or pain scales were not substituted for outcomes; rather, we directly visualized the articular cartilage. Nevertheless, imaging has shown promise in the classification and prediction of knee joint disease, so incorporating radiographic or MR data might serve as a promising next step [31–34]. This study is also notable for its use of SVMs in contrast to prior efforts which have solely used regression models or artificial neural networks. SVMs tend to outperform regressions in settings of low-dimensionality or match regressions in performance while using fewer variables [35]. Models that make use of deep learning, such as artificial neural networks, tend to have even more predictive power, but they provide little to no information about the utility of variables involved. Therefore, given the relatively small-to-intermediate number of variables considered and the modest prioritization of prediction over variable explanation, the current study employed SVMs—a current rarity in the field of OA prediction. Lastly, subjects in this study were randomly assigned to their treatment groups with stratifications for age and sex. Almost all OA outcome studies are case-controls or prospective cohorts, which require tight statistical monitoring for confounding—something that the structure of our study circumvents.

In conclusion, this study showed that synovial fluid and gait features early on after injury have the potential to predict later cartilage outcomes following traumatic injury to the ACL. More specifically, this preclinical analysis suggests that increased pressure on the injured leg combined with synovial fluid devoid of IL-2, IL-4, MMP-2, and MMP-12 is a possible indicator that cartilage is more at risk of becoming pathologic. If this finding is replicated in human patients, it has strong clinical implications for identifying at-risk individuals earlier on in their disease course so that care and rehabilitation can be more targeted and preventative. The utility of these features also implies their role in the pathogenesis of OA and justifies future studies to characterize their direct mechanisms of impact.

## Supporting information

S1 Appendix Detailed animal procedures. A summary of the surgical procedures and detailed information regarding animal husbandry and pain management.
(DOCX)

S2 Appendix Training performance of SVM models. (DOCX)

S3 Appendix Testing performance of SVM models. (DOCX)

S4 Appendix Cartilage sub-scores. (DOCX)

S1 File (R)

S2 File (PY)

S3 File (XLSX)

S4 File (CSV)
