# Supplementary material for: Predicting severity of cartilage damage in a post-traumatic porcine model: Synovial fluid and gait in a support vector machine
Source: PLoS One. 2022 Jun 8;17(6):e0268198. doi: 10.1371/journal.pone.0268198 (PMC9176756; doi:10.1371/journal.pone.0268198)
Supplement: S1 Appendix — A summary of the surgical procedures and detailed information regarding animal husbandry and pain management. (DOCX) [file pone.0268198.s001.docx]

**S1 Appendix:** Detailed Animal Procedures

**Animal model**

The adolescent Yucatan minipig model was selected for this study as it exhibits various features of human knee joints.^3^ The Yucatan minipig has been shown to develop macroscopic cartilage lesions consistent with posttraumatic osteoarthritis (PTOA) within one year following ACL transection.^1^ The cartilage damage typically develops in the medial compartment with more pronounced damage at areas adjacent to the tibial spine,^1^ consistent with the damage observed in human patients following ACL reconstruction surgery.^2^ Furthermore, the Yucatan minipig model has also been shown to develop other non-cartilaginous features of PTOA, such as an early synovitis along with accompanying changes in protein markers of extracellular matrix breakdown.^4^ The genetic^6^ and pharmacokinetic^5^ similarities between the porcine model and humans further support the use of the Yucatan minipig ACL transection model to study PTOA.

**Housing and husbandry**

Following delivery to the animal care facility, all animals underwent a minimum of a 7-day quarantine and stabilization period. The pigs were housed in single cages (a minimum of 22.5 ft^2^) with wood chips over the concrete floor. All pigs were housed in pens that were adjacent to pens housing other pigs. Pigs were allowed to ambulate at all times. Animals were fed at several scheduled times per day. However, food was withheld a minimum of 12 hours before surgery and before euthanasia. No animals were excluded from the study and no modifications to the approved protocol were necessary over the course of the study. The animals were not used in any previous study and were considered healthy via veterinarian examination upon arrival and the joints determined to be normal via intra-operative assessment.

**Anesthesia**

Anesthesia was induced using Telazol (4 mg/Kg) and Xylazine (2 mg/kg) followed by Propofol (3-7 mg/kg) and then maintained with Isoflurane (1-3 MAC) following intubation. Eyes were protected using an eye lubricant. Both limbs were shaved and scrubbed with Chlorhexidine and 70% alcohol until visibly clean, followed by a ten-minute evaporation period. Hoofs were covered with unsterile gloves. Animals were then transferred into the adjacent operating room, placed supine on a heating mat, and secured on the operation table. Animal health and anesthesia depth were maintained by monitoring respiratory rate, oxygen saturation, electrocardiogram, blood pressure, and body temperature. The surgical limb and ipsilateral lower body were then scrubbed three times using Betadine. Hoofs were covered with a sterile glove and secured using a sterile elastic wrap. One layer of sterile towels was placed around the surgical area, followed by a layer of sterile drapes, leaving only the surgical limb exposed during the procedure.

Prior to euthanasia, anesthesia was induced and maintained similar to that used for the surgical procedures. Animals were euthanized during deep anesthesia using an intravenous injection of a solution containing pentobarbital sodium and phenytoin sodium (Beuthanasia-D, 0.1ml/kg). Death was confirmed by a veterinarian technician by the absence of blood pressure and heart sounds prior to obtaining the tissue samples.

**Analgesia and Peri-operative care**

| **Drug** | **Dose** | | **Route** | **Frequency of application**  **(times/day)** | **Duration**  **(days)** |
| --- | --- | --- | --- | --- | --- |
|  | **mg/kg** | **ml** |  |  |  |
| Buprenorphine | 0.01 |  | Intramuscular | Once, pre-op | 1 |
| Fentanyl Patch | 2ug/kg/hr |  | Transdermal | Once, pre-op | 3 |
| Ceftiofur | 5 |  | Intramuscular | Once, pre-op | 1 |
| 0.5% Bupivicaine + 2% Lidocaine |  | 1.0 | Subcutaneous around wound | Once, post-op | 1 |
| Ondansetron | 4 |  | Intramuscular or Intravenous | Once, post-op | 1 |
| Tylenol elixir | 10-15 |  | Orally | Every 6 hours | As needed |

**Surgical Procedures**

*ACL transection*

A medial arthrotomy was created and the fat-pad partially resected to expose the ACL. The ACL was cut between the proximal and middle thirds of the ligament. A Lachman test was performed to verify complete transection. The knee was then irrigated with 500 cc of normal saline. For those animals assigned to receive no treatment, the incision was then closed in layers,^1^ and the ligament was allowed to heal naturally.

*ACL reconstruction*

Following ACL transection in the animals assigned to the ACL reconstruction group, fresh-frozen BPTB allografts, which were harvested from age, weight, and gender matched donors, were implnated as previously described.^1^ The entire patellar tendon (~10 mm in width) was used for the soft tissue portion of the graft while the bone plugs were trimmed to 7 mm diameter. Femoral graft fixation was achieved with a 6x20 mm bio-absorbable interference screw (Biosure; Smith & Nephew, Andover, MA). The graft was manually pre-conditioned in tension twenty times and firmly tensioned with the knee in maximal extension (~30^o^ for the pig). The distal block was secured in the tibia using a second 6 mm interference screw backed up with an extracortical tibial button. All incisions were closed in layers.

*Bridge-enhanced ACL repair*

For the animals assigned to the bio-enhanced ACL repair group, the repair was performed following ACL transection as previously described.^1^ In brief, an Endobutton carrying three looped sutures was passed thru a 4 mm femoral tunnel and flipped. Two of the sutures were threaded through the scaffold, into a predrilled tibial tunnel and fixed extracortically using a button with the knee in maximum extension. The remaining suture was tied to a Kessler suture of #1 Vicryl (Ethicon, Somerville, NJ), which had been placed in the tibial stump of the ACL. Three cubic centimeters of autologous blood were used to saturate and activate the scaffold. The scaffold-blood composite was allowed to set for a minimum of 10 minutes before completion. All incisions were closed in layers.

**References**

1. Murray MM, Fleming BC. Use of a bioactive scaffold to stimulate anterior cruciate ligament healing also minimizes posttraumatic osteoarthritis after surgery. *Am J Sports Med.* 2013;41(8):1762-1770.

2. Okafor EC, Utturkar GM, Widmyer MR, et al. The effects of femoral graft placement on cartilage thickness after anterior cruciate ligament reconstruction. *J Biomech.* 2014;47(1):96-101.

3. Proffen BL, McElfresh M, Fleming BC, Murray MM. A comparative anatomical study of the human knee and six animal species. *The Knee.* 2012;19(4):469-476.

4. Sieker JT, Ayturk UM, Proffen BL, et al. Immediate administration of intraarticular triamcinolone acetonide after joint injury modulates molecular outcomes associated with early synovitis. *Arthritis Rheumatol.* 2016;68:1637-1647.

5. Swindle MM, Makin A, Herron AJ, Clubb FJ, Jr., Frazier KS. Swine as models in biomedical research and toxicology testing. *Vet Pathol.* 2012;49(2):344-356.

6. Wernersson R, Schierup MH, Jorgensen FG, et al. Pigs in sequence space: a 0.66X coverage pig genome survey based on shotgun sequencing. *BMC Genomics.* 2005;6:70.
