# Supplementary material for: Predicting severity of cartilage damage in a post-traumatic porcine model: Synovial fluid and gait in a support vector machine
Source: PLoS One. 2022 Jun 8;17(6):e0268198. doi: 10.1371/journal.pone.0268198 (PMC9176756; doi:10.1371/journal.pone.0268198)
Supplement: S2 Appendix — (DOCX) [file pone.0268198.s002.docx]

**S2 Appendix**: Training Performance of SVM models.

**
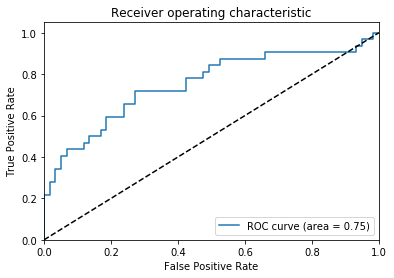

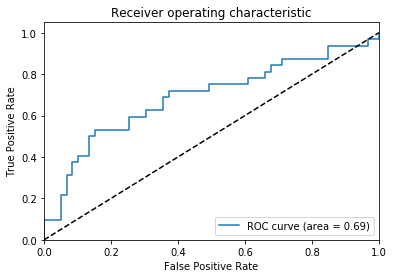
**

GEE

SF + Gait

Gait

SF

**
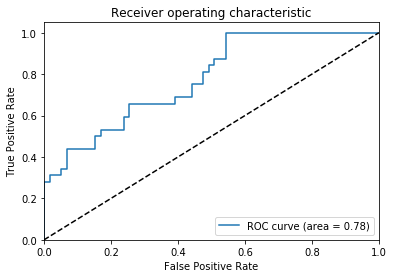
**
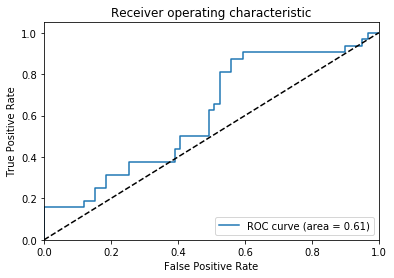


**Figure S2-1**: Receiver operating characteristic curves for the training performances of the four SVM models made in this analysis.

GEE

SF + Gait

Gait

SF


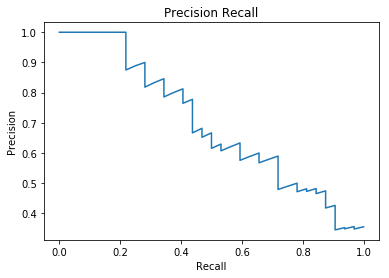

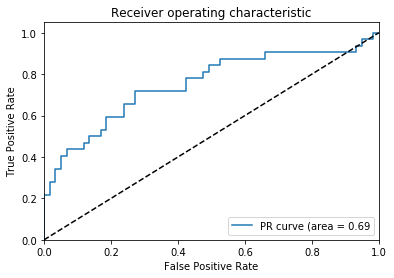

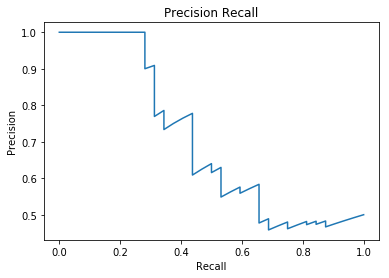

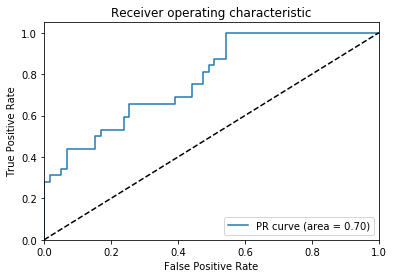

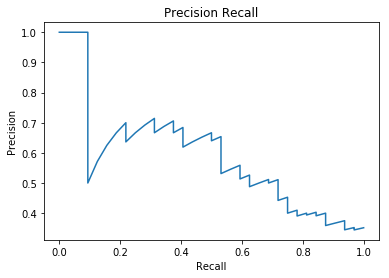

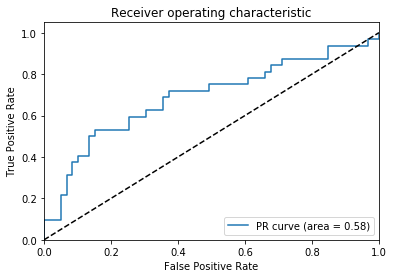

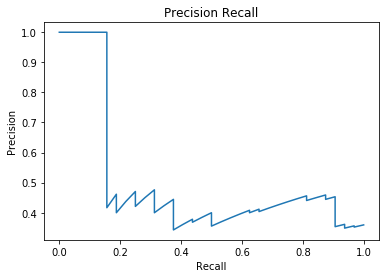

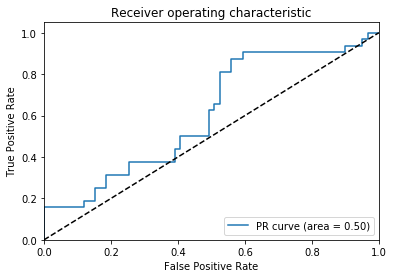


**Figure S2-2**: Precision Recall curves for the training performances of the four SVM models made in this analysis.

Truth (0 = Bad, 1 = Good)

GEE


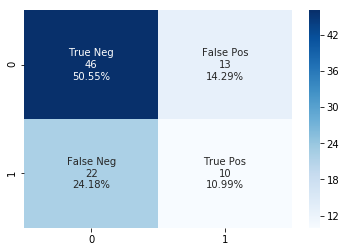

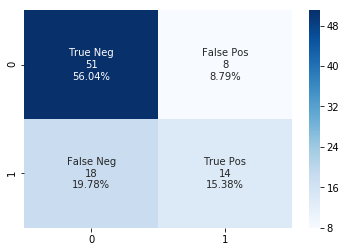

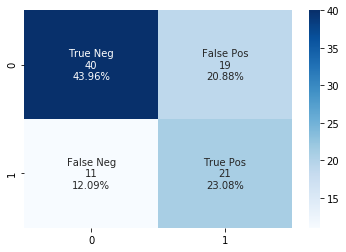

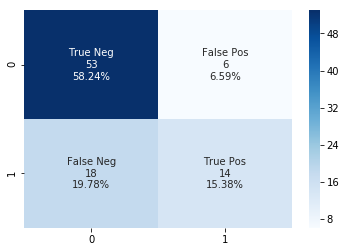


SF

SF + Gait

Gait

Prediction (0 = Bad, 1 = Good)

**Figure S2-3**: Confusion matrices for the training performances of the four SVM models made in this analysis.
