# Supplementary material for: Predicting severity of cartilage damage in a post-traumatic porcine model: Synovial fluid and gait in a support vector machine
Source: PLoS One. 2022 Jun 8;17(6):e0268198. doi: 10.1371/journal.pone.0268198 (PMC9176756; doi:10.1371/journal.pone.0268198)
Supplement: S3 Appendix — (DOCX) [file pone.0268198.s003.docx]

**S3 Appendix**: Testing Performance of SVM models.

**
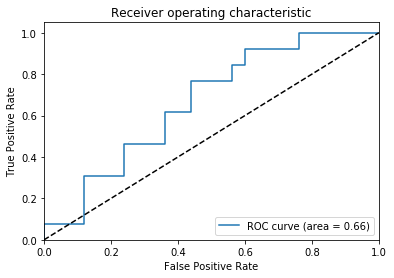

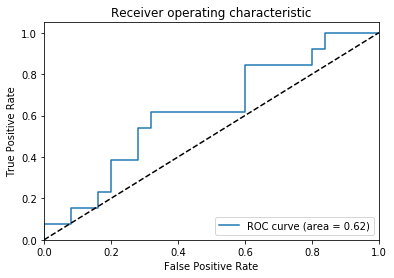

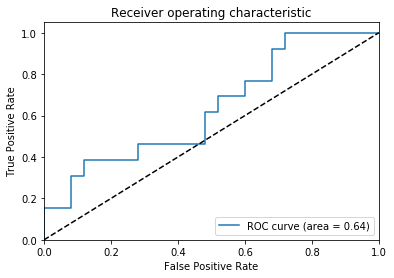

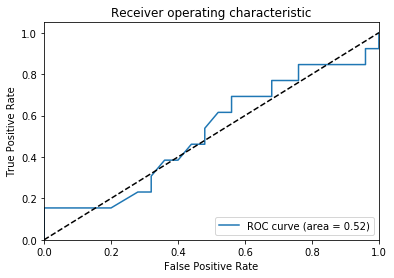
**

Gait

SF

SF + Gait

GEE

**Figure S3-1**: Receiver operating characteristic curves for the testing performances of the four SVM models made in this analysis.

**
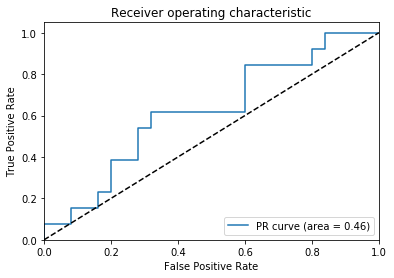
**
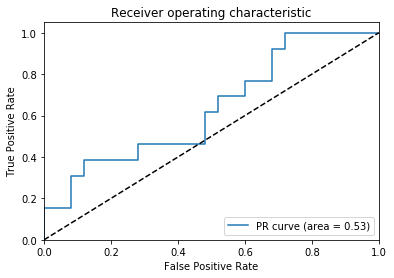

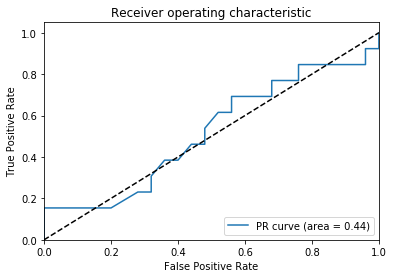
**
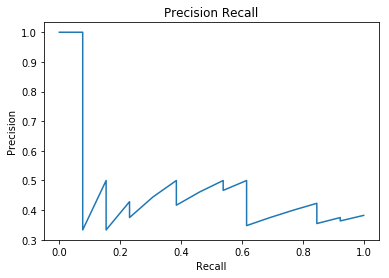
**
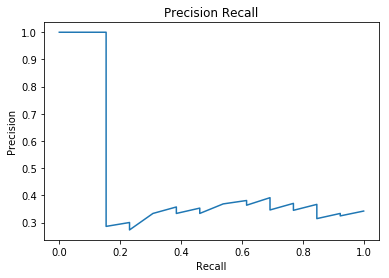
**
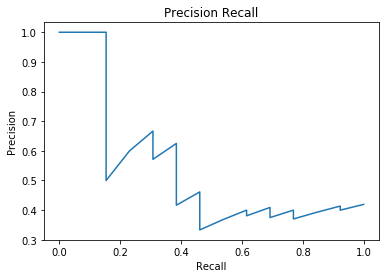
**

GEE


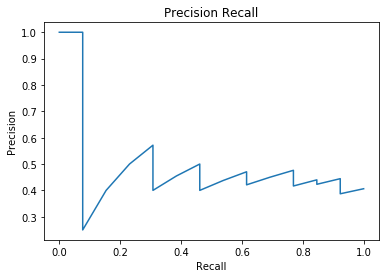

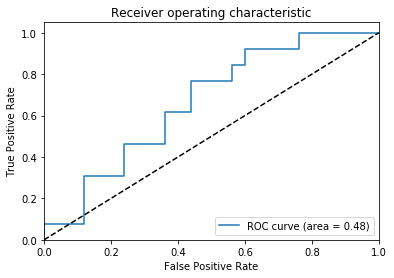


SF + Gait

Gait

SF

**Figure S3-2**: Precision Recall curves for the testing performances of the four SVM models made in this analysis.

Gait

**
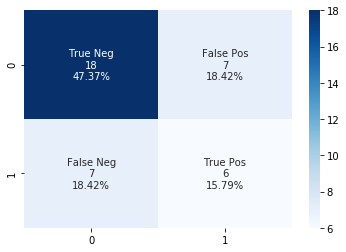
**

SF + Gait

GEE

Truth (0 = Bad, 1 = Good)

SF

**
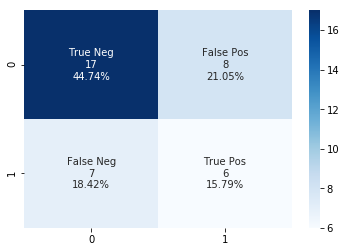

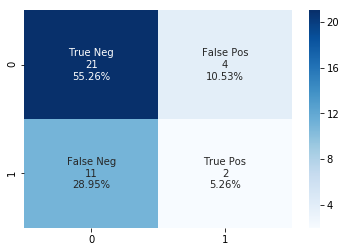
**

Prediction (0 = Bad, 1 = Good)

**
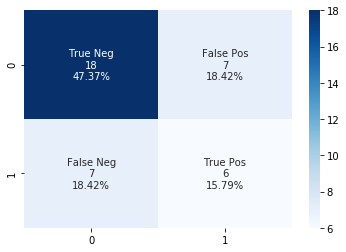
**

**Figure S3-3**: Confusion matrices for the testing performances of the four SVM models made in this analysis.
