# Supplementary material for: Predicting severity of cartilage damage in a post-traumatic porcine model: Synovial fluid and gait in a support vector machine
Source: PLoS One. 2022 Jun 8;17(6):e0268198. doi: 10.1371/journal.pone.0268198 (PMC9176756; doi:10.1371/journal.pone.0268198)

**S4 Appendix**: Frequency of macroscopic Sub-scores for the six compartments. MFC = medial femoral condyle, LFC = lateral femoral condyle, MTP = medial tibial plateau, and LTP = lateral tibial plateau.


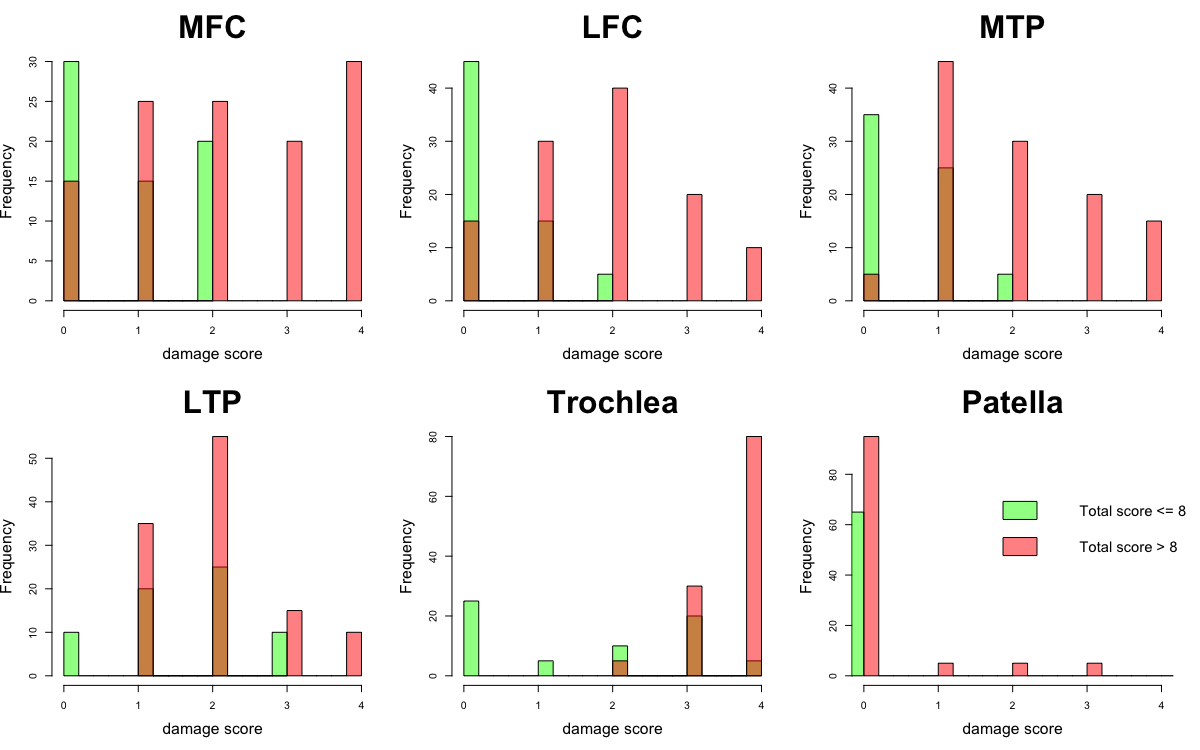

Supplement: S4 Appendix — (DOCX) [file pone.0268198.s004.docx]
